# Atorvastatin-induced Lichenoid Drug Eruption: A Case Report and Review of Statin-associated Cutaneous Adverse Events

**DOI:** 10.7759/cureus.7155

**Published:** 2020-03-01

**Authors:** Parnia Forouzan, Ryan R Riahi, Philip R Cohen

**Affiliations:** 1 Dermatology, University of Texas Medical School, Houston, USA; 2 Dermatology, DermSurgery Associates, Sugar Land, USA; 3 Dermatology, San Diego Family Dermatology, National City, USA

**Keywords:** adverse, atorvastatin, cutaneous, drug, lichen, lichenoid, eruption, planus, skin, statin

## Abstract

Statin medications [3-hydroxy-3-methylglutaryl coenzyme A (HMG-CoA) reductase inhibitors] are generally used to treat hypercholesterolemia. Lichenoid drug eruptions are a potential cutaneous side effect of medications including antibiotics, antimalarials, and statins. This drug eruption can mimic features of idiopathic lichen planus in clinical presentation and pathology. We describe the case of a 73-year-old man who developed a lichenoid drug eruption secondary to atorvastatin. His clinical features, in addition to histological findings, helped to establish the diagnosis. The cutaneous eruption resolved one month after the cessation of atorvastatin and with corticosteroid therapy. Statins have been associated with adverse events including bullous dermatosis, eosinophilic fasciitis, lichenoid drug eruption, and phototoxicity. Lichenoid drug eruption associated with statin therapy requires discontinuation of the statin medication; an alternative class of medication for the treatment of hypercholesterolemia is usually necessary.

## Introduction

Atorvastatin, a 3-hydroxy-3-methylglutaryl coenzyme A (HMG-CoA) reductase inhibitor, is commonly used to manage hypercholesterolemia. Atorvastatin usually prevents the production of cholesterol and other sterol products, including corticosteroids, vitamin D, and sex steroids, in the mevalonate pathway. However, statins can have a diverse array of effects beyond lowering the risk of cardiovascular disease [1]. Statins have been associated with various adverse cutaneous side effects including alopecia, bullous dermatosis, and lichenoid drug eruptions [1-18]. Lichenoid drug eruptions clinically mimic idiopathic lichen planus [19].

We report the case of a man with atorvastatin-induced lichenoid drug eruption. In addition, we describe the clinical and histopathologic characteristics of idiopathic lichen planus and lichenoid drug eruptions as well as cutaneous adverse reactions observed with statin medications.

## Case presentation

A 73-year-old man presented with a pruritic rash of two months' duration on his arms, chest, and neck. His past medical history was significant for asthma, erectile dysfunction, gastroesophageal reflux disease, and hypercholesterolemia. His current medications included atorvastatin, omeprazole, ranitidine, sildenafil, and Singulair (Merck & Co, Kenilworth, NJ). He had previously been seen by another physician who had topically treated him for eczema with betamethasone dipropionate 0.05% cream and crisaborole 2% ointment twice daily. His dermatitis had persisted despite therapy and he subsequently obtained a second opinion.

**Figure 1:**
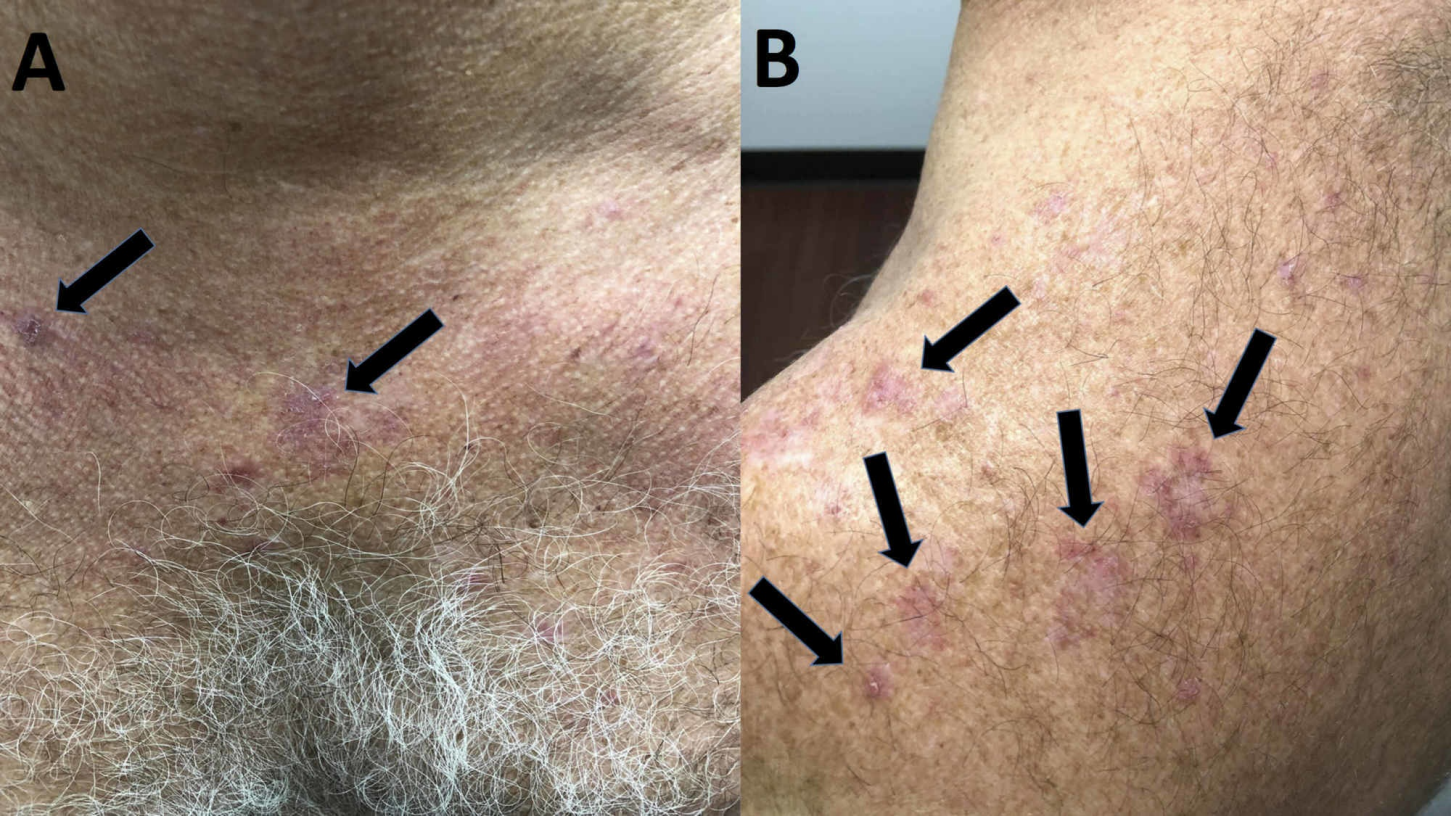
Cutaneous presentation of atorvastatin-induced lichenoid drug eruption Erythematous, pruritic plaques (black arrows) on the chest (A), neck, and the upper back (B)

**Figure 2:**
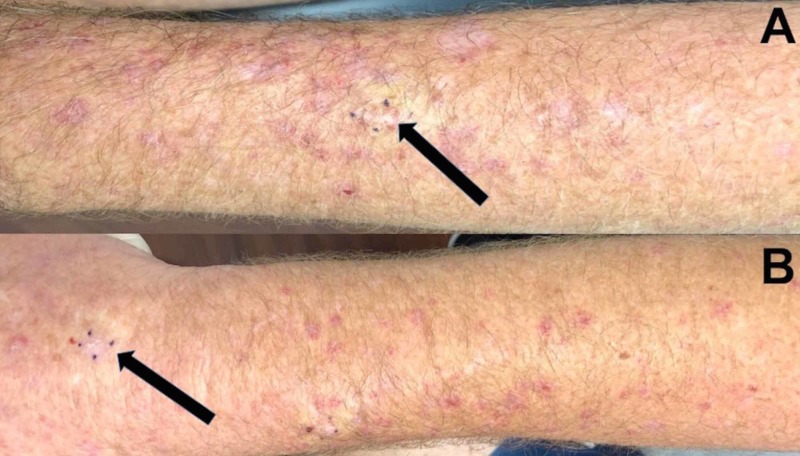
Skin biopsy sites of statin-induced lichenoid drug eruption on forearms A horizontal view of the biopsy sites (black arrows) of lichenoid drug eruption that presented as red, planar plaques on the left (A) and the right (B) forearms are each outlined by four small purple dots

Cutaneous examination revealed erythematous to purple scaly plaques on the bilateral forearms, chest, upper back, and neck (Figure 1). A shave biopsy of skin eruptions on both the left and right forearm was performed (Figure 2).

Microscopic examination revealed orthokeratosis, acanthosis, and spongiosis. A dense, band-like inflammatory infiltrate composed predominantly of lymphocytes was present in the upper dermis and along the dermoepidermal junction. In addition, apoptotic cells, eosinophils, and histiocytes were observed.

Pathologic findings pointed to lichenoid dermatitis with eosinophils. Correlation of the clinical history, lesion morphology, and pathologic findings established a diagnosis of a lichenoid drug eruption. We suspected that the causative agent was atorvastatin, which the patient had begun taking two months prior to the onset of his eruption.

Management included discontinuing the atorvastatin and treatment with prednisone, initially 40 mg daily with a gradual tapering of the dosage over 20 days. Additionally, a topical betamethasone dipropionate 0.05% cream to be applied twice daily for three weeks was also prescribed. His symptoms and skin eruption completely resolved and had not recurred at a one-month follow-up.

## Discussion

Adverse cutaneous events are a consequence of various medications including antibiotics, anticonvulsants, and statins. Earlier studies have observed that the majority of lichenoid drug eruptions were caused by either antimalarial agents or oral gold therapy [19].

The duration and onset of lichenoid drug eruptions are often dependent on the causative agent and dosage. Lichenoid drug eruptions occur most often in individuals between the age of 57 to 66 years and can have an average latent period of one year between the beginning of the medication treatment and the onset of an eruption [19]. This medication-induced eruption should be considered when an individual receiving statin treatment develops new lesions akin to lichen planus.

The clinical presentation and pathology of lichenoid drug eruptions can mimic those of lichen planus (Table 1) [15-16,19-20]. Both conditions present as erythematous to purple papules and plaques; however, lichenoid drug eruptions may be scaly, more pruritic, and resolve with greater residual hyperpigmentation [15,19]. In addition, Wickham’s striae (a lacy, white network of streak often located bilaterally on the buccal mucosa) and involvement of other mucosal areas are observed less frequently in drug-induced lesions [15,19]. Compared to the flexor surface distribution on extremities seen with idiopathic lichen planus, lichenoid drug eruptions may present in a photodistributed or symmetric pattern [15].

**Table 1:** Comparison between lichen planus and lichenoid drug eruption

Characteristic	Lichen planus	Lichenoid drug eruption	Reference
Morphology	Erythematous, planar, and polygonal papules are commonly described	Similar to lichen planus but can be scaly and more pruritic; alopecia, desquamation, eczematous papules, and greater residual hyperpigmentation may also occur	[15,19]
Pathology	A band-like lymphocyte infiltrate along the dermoepidermal junction is present along with apoptotic keratinocytes (Civatte bodies)	Similar to lichen planus but can also present with an infiltrate containing eosinophils. Focal parakeratosis, more prominent perivascular inflammation, and irregular granular layers may be present	[19,20]
Onset	Variable	Can appear one year after starting the causative medication; onset can vary based on the medication and dosage	[19]
Dermatology (primary lesion location)	Extremities	Arms, legs, and trunk	[15,19]
Distribution	Flexor surface	Symmetric, photodistributed pattern	[15,19]
Wickham’s striae	Commonly present	Typically not present	[15]
Oral/mucosal involvement	Majority of cases	Less common	[19]
Associated conditions	Diabetes mellitus, dyslipidemia, hepatitis B virus infection, hepatitis C virus infection, and thyroid dysfunction	Antimalarials, beta-blockers, oral gold therapy, penicillamine, statins, and thiazides	[16,19]
Prognosis	May spontaneously resolve	Less likely to spontaneously resolve and may not regress for months even after stopping the causative agent	[19]
Treatment	Can resolve spontaneously; however, oral and topical corticosteroids usually expedite resolution	May resolve after discontinuing the causative drug; however, oral and/or topical corticosteroids are usually needed to resolve the eruption	[19]

Microscopically, both lichenoid drug eruptions and idiopathic lichen planus exhibit a band-like lymphocytic infiltrate along the dermal-epidermal junction and apoptotic keratinocytes. Both conditions also show acanthosis, hypergranulosis, and hyperkeratosis [20]. However, an infiltrate with eosinophils in the dermis can help delineate lichenoid drug eruption from lichen planus [20].

Lichenoid drug eruptions are associated with medications. In contrast, lichen planus can be associated with systemic conditions such as diabetes mellitus and hepatitis B or hepatitis C viral infections. Lichenoid drug eruptions are also less likely to spontaneously resolve and may require discontinuation of the causative agent in addition to topical and/or oral corticosteroid therapy.

Several cutaneous adverse events have been described in patients who have received statins (Table 2) [1-18]. Among these, bullous dermatosis, cutaneous lupus erythematosus, dermatomyositis, eosinophilic fasciitis, and photosensitivity are the most common [1,3,5-6]. Acute generalized exanthematous pustulosis, alopecia, cheilitis, chronic actinic dermatitis, dermatographism, eczema, erythema multiforme, pityriasis lichenoides chronica, pityriasis rubra pilaris, porphyria cutanea tarda, purpuric lesions, and skin ulcers have also been associated with statin use [1-2,4,7-12].

**Table 2:** Cutaneous adverse events observed with statin medications CR: current report

Statin-associated adverse skin effects	Reference
Acute generalized exanthematous pustulosis	[1]
Alopecia	[2]
Angioedema	[1]
Bullous dermatosis	[3]
Cheilitis	[4]
Chronic actinic dermatitis	[1]
Cutaneous lupus erythematosus	[5]
Dermatographism	[1]
Dermatomyositis	[6]
Eczema	[1]
Eosinophilic fasciitis	[1]
Erythema multiforme	[7]
Ichthyosis	[1]
Lichenoid drug eruptions	[13-18, CR]
Lichen planus pemphigoides	[1]
Phototoxicity	[1]
Pityriasis lichenoides chronica	[8]
Pityriasis rubra pilaris	[9]
Porphyria cutanea tarda	[10]
Purpuric lesions	[11]
Skin ulcers	[12]
Toxic epidermal necrolysis	[1]

Lichenoid drug eruptions have historically been associated with antimalarials, gold, and penicillamine. More recently, they have been observed with antineoplastics, beta-blockers, and thiazides [16]. Our patient developed a lichenoid drug eruption secondary to atorvastatin. In addition to atorvastatin, other statin medications have also been implicated with lichenoid drug eruptions (Table 3) [13-18].

**Table 3:** Characteristics of patients with statin-induced lichenoid drug eruptions CR: current report

Drug, dosage	Age, race, and sex of patient	Location and onset	Morphology	Pathology	Treatment and result	Reference
Atorvastatin, 40 mg/day	73-year-old Caucasian male	Bilateral arms, chest, back, and neck; onset after two months on atorvastatin	Erythematous to purple, scaly patches	Lymphocytic infiltrate along the dermoepidermal junction with eosinophils and histiocytes	Discontinued atorvastatin; betamethasone and prednisone treatment; remission in one month	[CR]
Fluvastatin, 20 mg/day and lovastatin, 20 mg/day	59-year-old woman of unknown ethnicity	Extremities; onset after four weeks on fluvastatin. Redeveloped after two weeks on lovastatin	Papules and plaques with Wickham’s striae on papules. Some oral involvement was reported	A band-like lymphocytic infiltrate with apoptotic keratinocytes, hyperkeratosis, and vacuolar alteration	Discontinued fluvastatin use and treatment with mometasone-furoate resolved the initial eruption in three weeks; later treatment with lovastatin resulted in similar eruptions. Discontinued lovastatin; remission in three weeks	[13]
Pravastatin, unknown dosage	64-year-old woman of unknown ethnicity	Face and upper back; onset three months after beginning statin treatment	Dense freckling with no rash	Lymphocytic inflammation found along the dermoepidermal junction with basal cell damage and Civatte bodies	Discontinued statin; pigmentation resolved after nine months	[14]
Pravastatin, 10 mg/day	75-year-old Black man	Photodistributed, symmetric fashion on arms and hands; onset three weeks after beginning statin treatment. Reappeared after two weeks with pravastatin rechallenge	Erythematous plaques and papules with shiny scales	Focal hypergranulosis, hyperkeratotic stratum corneum, lymphocytic infiltrate, and vacuolar degeneration	Treatment with fluocinonide 0.05% gel and mupirocin 2% ointment was not effective. Discontinued statin; the eruptions healed after four weeks; rechallenge with pravastatin led to identical plaque formation	[15]
Rosuvastatin, 10 mg/day	65-year-old woman of unknown ethnicity	Trunk and extremities; onset three months after beginning statin treatment	Flat-topped and erythematous papules	A lymphocytic infiltrate was reported in the dermis with apoptotic keratinocytes and focal parakeratosis in the epidermis	Discontinued statin; treated with psoralen and ultraviolet A radiation therapy and with oral corticosteroid therapy. Remission in six months	[16]
Rosuvastatin, 10 mg/day and simvastatin, 10 mg/day	55-year-old South Asian woman	Right thigh with onset one week after beginning rosuvastatin; eruptions on her right thigh, back, and oral mucosa were reported at one-month follow-up	An erythematous rash	Apoptotic keratinocytes, basal vacuolar changes, and focal parakeratosis were present	Discontinued rosuvastatin; treatment with clobetasol propionate 0.05% cream. Remission in two months	[17]
Simvastatin, 10 mg/day	57-year-old woman of unknown ethnicity	Wrists, elbows, and buccal mucosa; onset after one month of statin use	Red papules and Wickham’s striae were noted	A lymphocytic infiltrate with eosinophils and histiocytes were reported. Compact orthokeratosis and focal parakeratosis in epidermis were found; Civatte bodies and vacuolar degeneration were also noted	Therapy with topical diflucortolone 0.1% cream did not resolve the eruption. Discontinued simvastatin and bezafibrate therapy; eruption began to resolve within four weeks, but the mucosal lesions persisted at the six-month follow- up	[18]

Lichenoid drug eruptions have been reported in one patient taking pravastatin 10 mg/day, two patients taking rosuvastatin 10 mg/day, and two patients on simvastatin at 10 mg/day [15-18]. Our patient with the atorvastatin-induced lichenoid eruption was being treated at 40 mg/day. Another patient developed a lichenoid drug eruption with pravastatin; however, this patient’s dosage was not stated [14]. Another patient developed a lichenoid drug eruption on fluvastatin 20 mg daily; when she switched to lovastatin 20 mg daily, she redeveloped this drug-induced eruption [13].

To the best of our knowledge, lichenoid drug eruptions secondary to statin medications have been reported in two men and five women including our patient [13-18]. These individuals ranged in age from 55 to 75 years with a median onset age of 64 years [13-18]. The median onset age was 74 years for men and 59 years for women [13-18]. Four of the patients were of unknown ethnicity; however, a Black man, a Caucasian man, and a South Asian woman were described [15,17]. In the individuals who experienced a statin-induced lichenoid drug eruption, the onset of the eruption ranged from 2 to 12 weeks after starting the statin medication with a median of four weeks [13-18].

The cutaneous adverse event appeared on the trunk and extremities in six patients; one of the patients had skin lesions that developed on the face [13-18]. Six patients presented with lichen planus-like violaceous papules, and one patient demonstrated dense freckling on her face [13-18]. Oral involvement was reported in three of the individuals, and Wickham’s striae were observed in two patients [13,17-18].

Histologic evaluation of the statin-induced lichenoid drug eruptions demonstrated lymphocytic infiltration of the dermoepidermal junction similar to idiopathic lichen planus [13-18]. Focal parakeratosis was reported in three patients [16-18]. Eosinophils were noted in two patients, including ours [18]. Hyperkeratosis was also noted in two patients’ statin-induced lichenoid eruptions [13,15].

Management of statin-induced lichenoid drug eruptions includes discontinuation of the causative statin agent and treatment with topical and/or oral corticosteroids. Six of the seven patients’ skin lesions, including ours, resolved with cessation of the statin medication and additional therapy: an oral corticosteroid, a topical corticosteroid, or both [13,15-18]. In some instances, the eruption persisted for several months after discontinuing the instigating agent. Indeed, with or without additional treatment, the statin-induced drug eruptions resolved within three weeks to nine months after the causative drug was stopped [13-18].

## Conclusions

Lichenoid drug eruptions share several features with lichen planus. However, unique characteristics of these drug-induced eruptions (including delayed onset, absence of Wickham’s striae, and presence of eosinophils microscopically) can help distinguish lichenoid drug eruptions from idiopathic lichen planus. Statins are generally used in the management of hypercholesterolemia; however, several adverse cutaneous events have been observed in patients treated with statins. Lichenoid drug eruptions are an uncommon adverse cutaneous event associated with statin medications. The new onset of lichenoid dermatitis in an individual receiving statin therapy should raise the concern that this skin eruption may be associated with the medication.
